# Heavy Metal Detection and Removal by Composite Carbon Quantum Dots/Ionomer Membranes

**DOI:** 10.3390/membranes14060134

**Published:** 2024-06-06

**Authors:** Emanuela Sgreccia, Francia Sarhaly Gallardo Gonzalez, Paolo Prosposito, Luca Burratti, Michele Sisani, Maria Bastianini, Philippe Knauth, Maria Luisa Di Vona

**Affiliations:** 1 Department of Industrial Engineering, University of Rome Tor Vergata, 00133 Roma, Italy; franciasarhaly.gallardogonzalez@students.uniroma2.eu (F.S.G.G.); prosposito@roma2.infn.it (P.P.); 2 Faculty of Science, Technology and Innovation of the University “Mercatorum”, 00186 Rome, Italy; luca.burratti@uniroma2.it; 3 R & D Department, Prolabin & Tefarm S.r.l., 06134 Perugia, Italy; michele.sisani@prolabintefarm.com (M.S.); maria.bastianini@prolabintefarm.com (M.B.); 4 CNRS, MADIREL (UMR 7246) and International Laboratory: Ionomer Materials for Energy, Aix Marseille University, Campus St Jérôme, 13013 Marseille, France; philippe.knauth@univ-amu.fr

**Keywords:** SPEEK, cadmium and lead removal, regeneration

## Abstract

The combination of ion exchange membranes with carbon quantum dots (CQDs) is a promising field that could lead to significant advances in water treatment. Composite membranes formed by sulfonated poly(ether ether ketone) (SPEEK) with embedded CQDs were used for the detection and removal of heavy metal ions, such as lead and cadmium, from water. SPEEK is responsible for the capture of heavy metals based on the cation exchange mechanism, while CQDs detect their contamination by exhibiting changes in fluorescence. Water-insoluble “red” carbon quantum dots (rCQDs) were synthesized from p-phenylenediamine so that their photoluminescence was shifted from that of the polymer matrix. CQDs and the composites were characterized by several techniques: FTIR, Raman, UV/VIS, photoluminescence, XPS spectroscopies, and AFM microscopy. The heavy metal ion concentration was analyzed by inductively coupled plasma–optical emission spectroscopy (ICP-OES). The concentration ranges were 10.8–0.1 mM for Pb^2+^ and 10.0–0.27 mM for Cd^2+^. SPEEK/rCQDs showed a more pronounced turn-off effect for lead. The composite achieved 100% removal efficiency for lead and cadmium when the concentration was below a half of the ion exchange capacity of SPEEK. The regeneration of membranes in 1 M NaCl was also studied. A second order law was effective to describe the kinetics of the process.

## 1. Introduction

Ion exchange membranes (IEMs) are composed of a polymer matrix that provides mechanical support and stability with functional groups that can exchange ions. IEMs are selective barriers that allow ions to pass based on their charge and size. When immersed in aqueous solutions, nanoscale channels are formed that allow IEMs to interact with heavy metal ions, capturing and exchanging them with toxicologically safe ions [1,2,3]. This type of technology results in an effective, environmentally friendly, and reusable way to remove heavy metals from water [4].

Heavy metals in water are considered a significant hazard since prolonged exposure to them can lead to several environmental and health problems, including neurological disorders, development issues, and increased risk of cancer. Two specific metal ions are the focus of this research: lead (Pb^2+^) and cadmium (Cd^2+^). The values established as permissible by the World Health Organization on drinking water are 0.01 and 0.003 ppm, respectively, for lead and cadmium [5]. Nowadays, 71% of Pb is used in batteries, 12% in pigments, 6% in ammunition, and 3% in cable sheeting [6]. Cadmium is a common impurity in zinc and lead ores; its applications go from the production of coatings, pigments and alloys to battery electrodes and photocatalysts. Nonetheless, it is considered the most toxic heavy metal found in industrial effluents [6].

Carbon quantum dots (CQDs) stand out by exhibiting unique optical and electronic properties thanks to their quantum confinement effect [7]. With applications in imaging, solar fuels, and sensors, CQDs are environmentally friendly and have a customizable surface chemistry, contributing to their appeal in cutting-edge technologies across various domains [8].

The fluorescence of CQDs is believed to arise from a combination of different origins, such as quantum confinement, surface functional groups, defect states, and conjugated π-electron systems. Despite the progress made in understanding the fluorescence mechanism of CQDs, the exact interplay between these factors and their relative contributions to the overall fluorescence remain areas of active research [9,10]. Furthermore, the optical properties of CQDs can be influenced by their synthesis, including the temperature and duration of the heat treatment, because these parameters can affect the size, structure, and surface chemistry of the CQDs [11,12].

CQDs are reported to reveal different heavy metals like Hg, Zn, Cd, Cu, Fe, and Pb, and their study is focalized on the sensing properties [11,13,14,15,16,17,18]. This type of sensing probe now has applications in monitoring mode but does not focus on heavy metal capture or water filtration [19,20]. Sometimes, CQDs are inserted in an inert polymeric matrix [21,22,23]; however, the combination of IEMs with CQDs has, to our knowledge, not been investigated before.

By incorporating CQDs into IEM arrays, detection and removal capabilities can be integrated into a single composite material. When water-containing heavy metal ions pass through the composite membrane, functionalized CQDs can detect heavy metal contamination by exhibiting changes in fluorescence or other optical properties. The membrane selectively binds heavy metal ions while allowing other ions and molecules to pass through, effectively purifying the water. Furthermore, the addition of CQDs into the polymer matrix can provide extra functional groups (hydroxyl, carboxyl, amine), improving the performance of the ionomer. However, it is important to consider that the water solubility of small size CQDs may lead to a loss from the membrane during operation. Moreover, to improve their efficiency as optical sensors, CQDs should be chosen so that the peak of their photoluminescence is ‘far’ from that of the polymer matrix.

CQDs can be synthesized from various carbon sources using simple methods such as hydrothermal synthesis, microwave-assisted synthesis, or synthesis by pyrolysis, and the functionalization with specific ligands or receptors allows selective capture of heavy metal ions [24]. The properties of CQDs depend on the precursors used during the synthesis, and the synthesis type and parameters (time, temperature, etc.) chosen.

In this work, the IEM was sulfonated poly(ether ether ketone) (SPEEK), which was intensively investigated in our laboratory [2,3]. Water-insoluble, “red” carbon quantum dots (rCQDs), whose photoluminescence was energy-shifted from that of the polymer matrix, were synthesized from p-phenylenediamine that easily forms conjugated structures. The combination of ionomer and CQDs gives a material with simultaneous heavy metal detection and removal capability.

## 2. Materials and Methods

### 2.1. Synthesis of Red CQDs (rCQDs)

The synthesis was adapted from ref. [25]. p-Phenylenediamine (pPDA, 80 mg) was dissolved in distilled H_2_O (30 mL) at 30 °C. Afterwards, orthophosphoric acid 85% (H_3_PO_4_, oPA, 0.75 mL) was added, and the mixture remained under agitation at room temperature for 10 min (mol reagent ratio pPDA:oPA = 1:16). The solution was transferred into an autoclave, and it was kept at a constant temperature of 180 °C for 24 h. Following this time, the product was cooled down to room temperature to proceed to filtration with a KNF D-79112 vacuum pump using a polyamide membrane filter (0.2 μm) to remove the unreacted material. The remaining solution was neutralized with 1 M NaOH (10 mL). The neutralized product was centrifuged 3 times with a REMI Centrifuge NEYA-16. The synthesized CQDs were washed two times in water and centrifuged. No fluorescence was found in the water, indicating the insolubility of rCQDs. The CQDs were solubilized in 50/50 vol% EtOH/H_2_O. After the third centrifugation, the liquid phase was separated and stored at 4 °C.

### 2.2. Preparation of SPEEK/rCQDs Composite

Sulfonated poly(ether ether ketone) (SPEEK) was synthesized according to ref. [26]. rCQDs in EtOH/H_2_O (50/50 vol %) were dried at 40 °C under vacuum. The solid product (8 mg) was dissolved in 1 mL of DMSO and added to SPEEK (152 mg) in 5 mL of DMSO. The homogeneous solution was transferred to a Petri dish for a solution-casting procedure and heated at 80 °C for 48 h. After this time, the membrane was left to rehydrate at room temperature overnight and finally separated wholly from the Petri dish. The obtained membranes had 5 wt % of rCQDs with a thickness ranging from 50 to 60 μm.

### 2.3. Ion Exchange Capacity (IEC)

The as-prepared membranes were kept in 1.5 M NaCl solution for 48 h to induce the H^+^/Na^+^ ion exchange. This solution was back-titrated with NaOH 20 mM. The IEC was calculated following the parameters of ref. [26] and was 1.65 meq/g.

### 2.4. Water Uptake (WU)

The membranes were dried overnight at 50 °C. They were then transferred to a desiccator with P_2_O_5_ to reach room temperature. Quickly, they were weighed before being submerged in water for 24 h and weighed again after this procedure. The water uptake is obtained according to Equation (1), where *m_d_* and *m_w_* correspond to the dry and the wet mass of the membranes, respectively.

(1) $$WU/\%=\frac{m_{w}-m_{d}}{m_{d}}\times 100$$

### 2.5. Raman Spectroscopy

An Optosky ATR8300MP Raman Microscope with an excitation wavelength of 785 nm was used for this analysis. Two drops of rCQDs of the ready-to-use liquid part were set onto a silicon wafer and dried at 80 °C for 4 h. This process was repeated four more times to obtain a layer thick enough for analysis. Spectra were collected for at least 10,000 scans using laser powers of 75 and 85 mW. No appreciable difference in the spectra was observed.

### 2.6. Fourier-Transform Infrared Spectroscopy (FTIR)

FTIR analysis was performed using a Perkin Elmer Spectrum 2 IR spectrometer equipped with an ATR Zinc Selenide (ZnSe) crystal; the spectra were recorded in the transmission mode in the range of 4000–500 cm^−1^ with a resolution of 2 cm^−1^.

### 2.7. X-ray Photoemission Spectroscopy (XPS)

The samples for XPS analysis were prepared by depositing a drop of solution on a gold foil, then drying in air. Photoemission spectra were acquired using a spectrometer Escalab 250Xi (Thermo Fisher Scientific Ltd., Waltham, MA, USA) with a monochromatic Al Kα (1486.6 eV) excitation source. The binding energy (BE) scale was corrected by positioning the C 1s peak of aliphatic carbon (C-C, C-H bonds) at BE = 285.0 eV and controlling if the Fermi level corresponds to BE = 0 eV.

### 2.8. Atomic Force Microscopy (AFM)

Membrane surfaces were observed with a Nanosurf FlexAFM Microscope (Nanosurf, Liestal, Switzerland). The as-prepared membranes were attached to the surface of a silicon wafer with cyanoacrylate (superglue) and analyzed in a tapping mode with a BT06865 Dyn190Al cantilever tip having a radius below 10 nm, a nominal resonance frequency of 190 kHz, and a nominal force constant of 48 N/m.

### 2.9. UV/VIS/NIR Absorption Spectroscopy

The adsorption spectra of samples were recorded with a Lambda 750 Perkin Elmer UV/Vis/NIR spectrophotometer in the range 300–800 nm (PerkinElmer, Waltham, MA, USA). The liquid samples were measured in 1 mm quartz cuvettes, while membrane samples were recorded by fixing them on a quartz substrate. The solid rCQDs were dissolved in DMSO (3 mL) and analyzed.

### 2.10. Photoluminescence Spectroscopy

The photoluminescence of CQDs and the membranes was analyzed by a custom-made apparatus; the detailed scheme of the experimental set-up is reported in the Ref. [27]. A LED light was used as the excitation source (430 nm, power 60 μW). The excitation light impinged the sample with an angle of 40° with the respect to the normal of the sample surface. The emission was collected frontally by optical lens. To avoid the presence of the excitation wavelength inside the monochromator, a long-pass filter was placed in front of the monochromator slit. The emission of the membranes was measured at three different points of each piece of membrane, and the mean of the three was calculated to ensure trustworthy results.

### 2.11. Optical Microscopy

Optical micrographs were recorded with a Nikon Eclipse optical microscope equipped with objectives from 10× to 100× (Nikon, Tokyo, Japan).

### 2.12. Inductively Coupled Plasma–Optical Emission Spectroscopy (ICP-OES)

The concentrations Cd(II) and Pb(II) in the water were measured by ICP-OES (Avio 200, Perkin-Elmer, Waltham, MA, USA). Solutions were properly diluted and analyzed after a suitable calibration. The values reported of Cd(II) and Pb(II) correspond to an average of three independent measurements.

### 2.13. Batch Sorption Studies

Different concentrations of metals were prepared starting from Pb(NO_3_)_2_ and Cd(NO_3_)_2_. 4H_2_O. Before all measurements, the membranes were cut into several pieces, weighed, and immersed into 1 M NaCl solution for the adsorption of Pb(II) or 1 M Na_2_CO_3_ for Cd (II) and kept under stirring overnight. The membranes were then washed with deionized water and introduced into the heavy metals solutions (10 mL) under constant agitation during different periods of time. Subsequently, the membranes were removed, washed with water, and dried in a desiccator with P_2_O_5_. The sorption analysis was made by ICP-OES using a blank sample of the prepared metal solutions as a reference. The regeneration tests after the sorption of Pb(II) were made by immersion of the membranes in 1 M NaCl overnight followed by a quick wash in pure water.

### 2.14. Stability Test of Composite Membranes

To ensure that the membranes did not release CQDs during the water immersion, a piece of each membrane was cut and put into a test tube pre-filled with deionized water for 24 h. After that, the membranes were removed from the tubes, and the liquid was examined under UV light (365 nm) to verify the absence of any luminescence. Indeed, no luminescence was observed showing that the membrane did not release CQDs during use.

## 3. Results and Discussion

**Figure 1:**
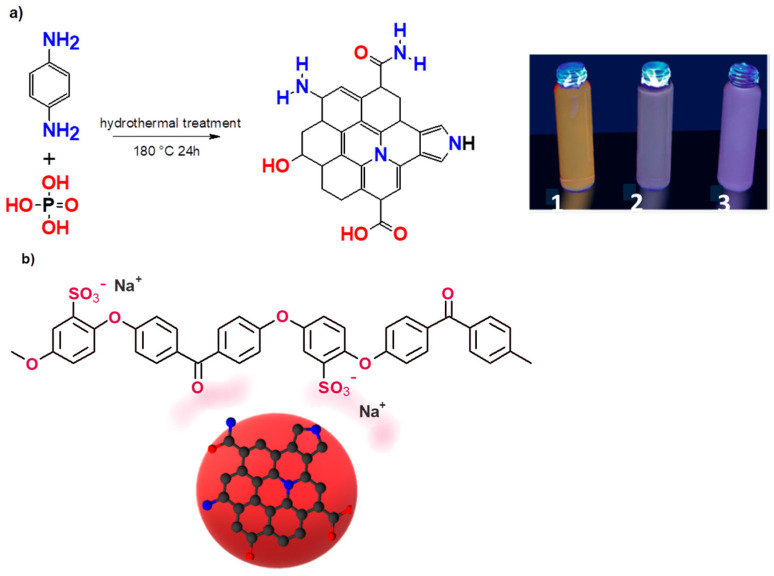
(**a**) Schematic representation of red CQDs. In the box, (1) after centrifugation, in water; (2) before the drying process, in 50 vol% EtOH/H_2_O; (3) dried and dissolved in DMSO. (**b**) Schematic representation of the SPEEK-CQD composite.

Figure 1a shows the synthesis of rCQDs and the hypothesized structure based on the characterization results. The composite is represented in Figure 1b, where the possible interaction among the hydrophilic groups of the polymer and the hydrophilic moieties of the CQDs are underlined, which avoid the loss of CQDs from the composite polymer. The figure also shows the interaction of the anionic groups of the polymer with the divalent metal cations.

**Figure 2:**
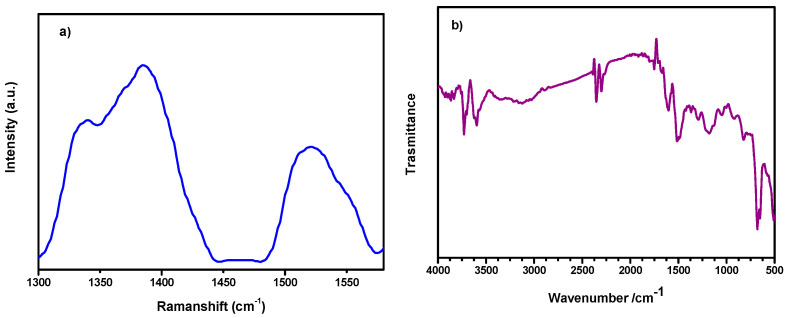
(**a**) Raman and (**b**) FTIR spectra of rCQDs.

Figure 2a displays the Raman spectrum of rCQDs. Two typical main bands appear in the spectrum at 1385 and 1523 cm^−1^. The first one is the D-Band, which is related to the defects of the graphitic lattice (sp^3^ carbon); the second, the G-band, is related to the sp^2^ carbon lattice (graphitic carbon). The intensity of the D-Band is therefore linked to “edge” carbon atoms, which introduce imperfections in the lattice, or more generally to structural modifications due to doping atoms; in the G-Band, the intensity is due to carbon atoms engaged in double bonds or aromatic rings. The intensity ratio (ID/IG) between the two peaks is an estimate of the disorder of the structure with respect to the graphitic lattice [28,29]. In our case, the ratio is 1.7, showing a relatively high amount of defects. [30]. Theoretically, CQDs emit red light due to their large size of sp^2^ domains; however, this large size always indicates a low amount of surface defects leading to a low quantum yield [31].

The FTIR spectrum of the rCQDs is reported in Figure 2b. The two peaks at 3710 and 3600 cm^−1^ are characteristic of primary aromatic amines/amides due to asymmetric and symmetric N-H stretching. The O-H bond vibrations appear around 3200 cm^−1^, and slight peaks at 2900 cm^−1^ are assigned at aliphatic C-H stretching. The spectrum displays the C=O stretching of the amide groups at 1680 cm^−1^ and the aromatic C=C stretching at 1608 cm^−1^ and 1500 cm^−1^. This latter band also overlaps with the bending vibration of pyridinium ions [32]. The signal at 1300 cm^−1^ is associated with C-N stretch, while the large band at 1200 cm^−1^ is ascribed to the C-O bends.

**Figure 3:**
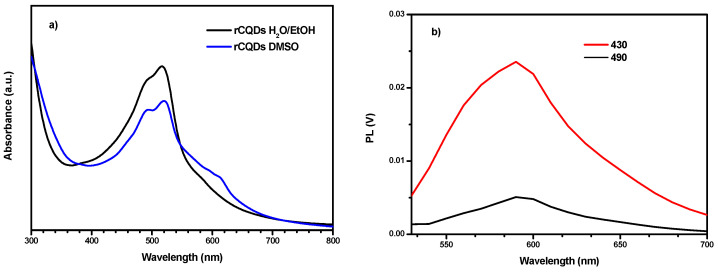
(**a**) UV-Vis absorption spectra of rCQD in EtOH/H_2_O and DMSO; (**b**) PL emission spectra of rCQD in EtOH/H_2_O after an excitation with wavelength at 430 and 490 nm.

Figure 3a shows the optical absorption spectra in the 300–800 nm range of the rCQDs in H_2_O/EtOH and in DMSO. The maximum adsorption was at 517 nm for the sample in H_2_O/EtOH and at 520 nm in DMSO, showing a small solvatochromic effect. Two different excitation wavelengths were selected for PL measurements of rCQDs in H2O/EtOH, namely 430 and 490 nm (Figure 3b).

When excited at 430 nm, the rCQDs show a higher emission intensity at 590 nm which corresponds to the orange color of the light spectrum, showing a Stokes shift [33] relative to the absorption band by approximately 70 nm. By using a 490 nm excitation, the photoluminescence intensity largely decreases. Nonetheless, the emission remains at the same wavelengths, following Kasha’s rule [34], since fluorescence only shows characteristics of the lower electronic state. For this reason, the 430 nm excitation wavelength was chosen for both CQDs and membranes.

**Figure 4:**
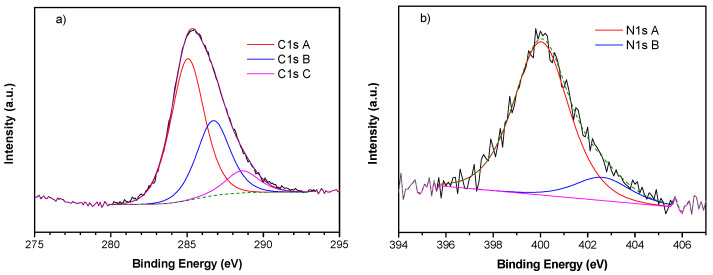
XPS spectra of (**a**) C 1s region and (**b**) N 1s region.

**Figure 5:**
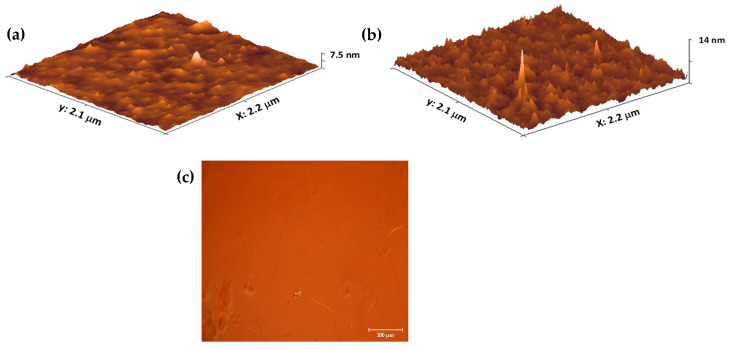
AFM images for (**a**) SPEEK and (**b**) SPEEK-CQD composite membrane, and (**c**) optical micrograph of a composite membrane.

Figure 4 shows the XPS spectra of the C and N atoms with the peak fitting. C is present in three components (Figure 4a): component A at 285.0 eV assigned to -C=C- and aliphatic carbon, component B at 286.7 eV due to -C=N and C-O bonds, and component C at 288.6 eV for carboxylic -C=O bonds [35,36]. Nitrogen (5 atom %, Figure 5b) is present as pyrrolic/amine/amide at 400 and pyridinic oxide at 402.6 eV [24].

A schematic figure of the SPEEK/rCQDs composite is represented in Figure 1b. The WU, and therefore the hydrophilicity, increased for the composite (WU = 166%) with respect to pure SPEEK (WU = 23%), due to the addition of extra functional groups on the surface of CQDs. It is well known that WU and IEC are correlated: the higher the IEC, the higher the WU. For water filtration, a large IEC may seem convenient to have a high sorption capacity and permeability; however, increasing the IEC too much can cause over-swelling, which leads to a loss in selectivity and/or full collapse of the membranes [37]. Since the membranes did not gel in water and no loss of CQDs was traced after immersion for 24 h in water, it was concluded that the degree of functionalization and the mass percentage of CQDs in the composites (5%) were appropriate.

The AFM images (Figure 5a,b) show pure SPEEK and composite membranes. The surfaces are free of pores larger than the cantilever tip diameter (<20 nm); the presence of the CQDs gives a slightly higher average roughness for the composite membrane (Rms = 7.5 nm for SPEEK and Rms = 14 nm for SPEEK-CQD).

A typical optical micrograph of the composite membrane is presented in Figure 5c. A relatively homogeneous distribution of the composite material can be clearly observed without visible micro and nano porosities.

**Figure 6:**
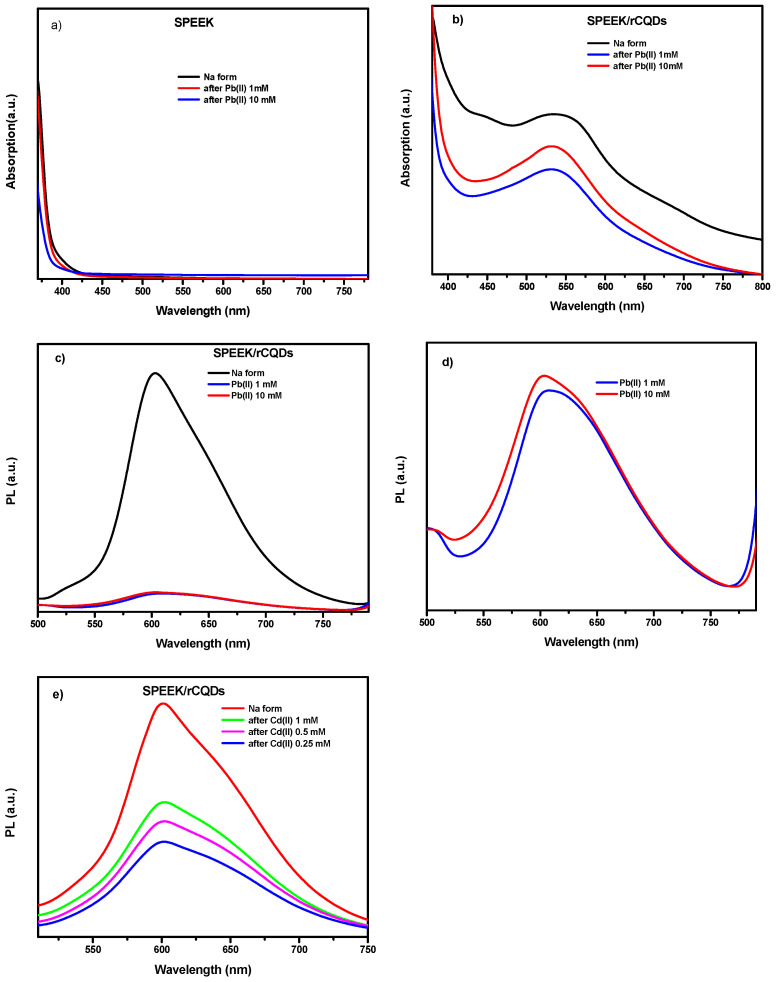
UV-Vis absorption spectra for (**a**) SPEEK and (**b**) composite membranes after immersion in Pb(II) solutions. PL emission spectra for (**c**) composite membranes before and after immersion in Pb(II) solutions; (**d**) magnification of (**c**); (**e**) composite membranes before and after immersion in Cd(II) solutions.

Figure 6 shows the absorption and PL emission spectra for both SPEEK and the red composite membranes exchanged with Na^+^ and after immersion in 1 and 10 mM Pb(II) for 24 h. For SPEEK (Figure 6a), no absorption peaks are noticeable. Xing et al. [38] demonstrate that SPEEK has absorption peaks in the range of 200–300 nm. When rCQDs are introduced into the SPEEK matrix (Figure 6b, black curve), the spectrum has a main peak at 560 nm and a shoulder at 430 nm. After the heavy metal exposure in both concentrations (1 and 10 mM), the peak at 430 disappears.

The same samples were also analyzed by emission spectroscopy to understand the luminescence properties of the red membranes at different stages of lead capture and to determine if they act as PL sensors when heavy metal ions are captured. Figure 6c depicts the PL spectra of the red composite membrane when stimulated at 430 nm excitation wavelength; the spectra were normalized by the optical absorption at 430 nm for direct comparison.

The red composite (Figure 6c) emits at 600 nm with a high intensity when sodium is present in the solution, while after Pb(II) capture, the emission signal decays and almost disappears (turn-off of emission) independently on the Pb ion concentrations. The sensitivity of the turn-off effect can be appreciated in the magnification of Figure 6d indicating that the number of active sites (CQDs) which are responsible for that behavior are completely saturated leading to a decrease in the PL signal. This behavior cannot be linked directly to the removal efficiency (RE), since most of the Pb ions are captured by the anionic groups of the SPEEK membrane, but is a clear indication of the presence of the contaminant captured by the membrane.

Figure 6e displays the results of the photoemission behavior of the composite analyzed for the capture of Cd(II) at different concentrations: 1, 0.5, and 0.25 mM. Also, in this case, a similar behavior can be appreciated even if a slight difference, dependent on the concentration of cadmium ions, is present, probably due to the different absorption of the red composite for the three concentrations.

Table 1 reports the results of the heavy metal ion sorption measurements. The removal efficiency (*RE*) was calculated using Equation (2).

(2) $$RE/\%=\frac{Ci-Cf}{Ci}\times 100\;$$

where *Ci* is the concentration before and *Cf* is the concentration after the metal ion sorption by the membrane.

Given the IEC of the SPEEK membrane (IEC = 1.65 meq/g), the amount of Pb or Cd that can be sorbed by 30 mg of membrane corresponds to 0.025 mmol. For larger quantities, RE is below 100%, because there are no more ion exchange sites available in the membrane (Table 1).

**Figure 7:**
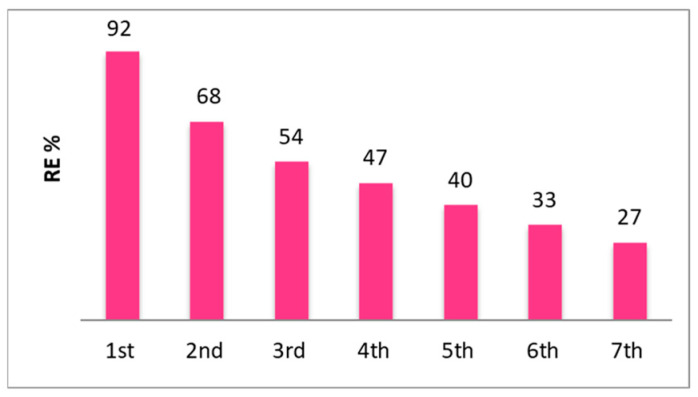
Regeneration test of the SPEEK-CQD composite membrane after 5 mM Pb(II) sorption for 7 cycles.

The regeneration behavior was studied in detail for the composite membrane exposed to 5 mM Pb(II). The exchange with sodium allowed the direct use of the regenerated membrane and avoided the use of other chemicals such as ethylenediaminetetraacetate (EDTA) [39]. The results for seven cycles of sorption/desorption of Pb(II) are shown in Figure 7. An asymptotic behavior is presumably observed, suggesting that some ion exchange sites are occupied by irreversibly adsorbed Pb^2+^. The preferential adsorption of lead vs. sodium is due to the larger ionic radius and larger charge of lead, which favors the interaction with the polarizable functional groups present in the composite [40].

Kinetic tests for lead and cadmium are summarized in Table 2.

The data can be described by a second order kinetic law, according to Equation (3):

(3) $$\frac{t}{c}=\frac{1}{kc{{}^{\circ}}^{2}}+\frac{t}{c{}^{\circ}}\;\;$$

**Figure 8:**
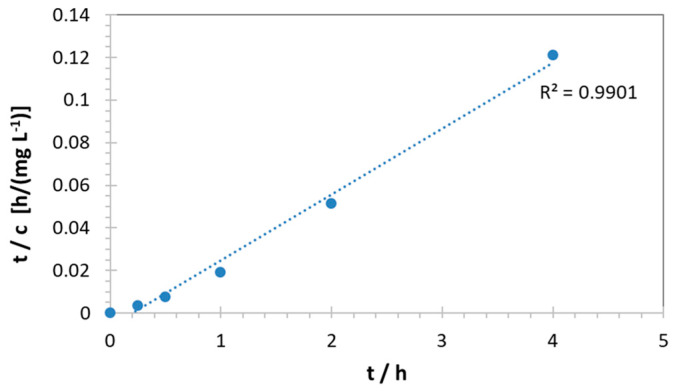
Second order kinetic plot of lead concentration vs. time.

Figure 8 presents the plot for the case of cadmium. It has been shown previously that second order kinetics are observed when the amount of the sorbed species is not negligible vs. the number of available sorption sites in a material [4].

## 4. Conclusions

Composite membranes containing a cation exchange polymer (sulfonated poly(ether ether ketone) and “red” carbon quantum dots, synthesized from the precursor p-phenylenediamine, were prepared.

The change in photoluminescence is a powerful tool for the detection of heavy metal ions, here lead and cadmium; the amount of cations sorbed depends on the ion exchange capacity of the ionomer, and a removal efficiency of 100% can be attained. The cation sorption kinetics can be described by a second order law. The regeneration of the IEM-CQD in 1 M NaCl solutions leads to a partial recovery of the sorption capacity; the irreversible loss of removal efficiency is probably due to the preferential sorption of lead and cadmium ions, related to their higher charge and size.

Overall, the integration of CQDs into composite IEMs offers a promising solution for water purification with simultaneous detection and removal of heavy metal ions, addressing both environmental monitoring and remediation needs.

## Figures and Tables

**Table 1:** Heavy metal removal by composite IEMs after 24 h.

Pb(II)		
Ci mg/L (mM)	Cf mg/L	RE%
2238 (10.8)	1253	44
1036 (5.0)	83	92
207 (1.0)	0	100
**Cd(II)**		
1130 (10)	346	41
125 (1.1)	3.8	97
63 (0.55)	0	100
31 (0.27)	0	100

**Table 2:** Kinetic data for Pb(II) and Cd (II).

Time (h)	Pb(II) Concentration/mg/L (mM)	RE (%)
0	145 (0.7)	0
0.25	116	20
0.5	109	33
1	89	39
2	75	48
4	55	62
	**Cd(II) concentration/mg/L (mM)**	
0	85 (0.7)	0
0.25	72	15
0.5	65	23
1	52	38
2	39	53
4	33	61

## Data Availability

The original contributions presented in the study are included in the article, further inquiries can be directed to the corresponding authors.
